# An Unusual Case of Ascending Pancreatitis with Mediastinal Involvement: A Case Report with CT and MRI Findings

**DOI:** 10.1155/2014/925105

**Published:** 2014-05-14

**Authors:** Ernesto Di Cesare, Alessandra Di Sibio, Antonio Gennarelli, Valentina Felli, Valentina Vellucci, Ines Casazza, Carlo Masciocchi

**Affiliations:** ^1^Division of Radiotherapy and Cardiac Radiology, Department of Biotechnology and Applied Clinical Sciences, San Salvatore Hospital, University of L'Aquila, L. Natali Street 1, 67100 L'Aquila, Italy; ^2^Division of Radiology, Department of Biotechnology and Applied Clinical Sciences, San Salvatore Hospital, University of L'Aquila, L. Natali Street 1, 67100 L'Aquila, Italy; ^3^Department of Radiology, Sant'Andrea Hospital, Sapienza University, Via di Grottarossa 1035/39, 00189 Rome, Italy

## Abstract

Fluid collections are common findings of pancreatitis and spread, more often, along preferential drainage pathways in the abdomen. In some rare cases, fluid collections may spread towards extra-abdominal sites like the mediastinum leading to the formation of mediastinal collections. We present the case of a 52-years-old man with pain in the right upper quadrant of the abdomen and mid-epigastrium lasting for some hours. Laboratory tests suggested a diagnosis of pancreatitis. CT and subsequent MRI revealed changes consistent with acute exacerbation on chronic pancreatitis spreading to the mediastinum and to the greater omentum. The patient received medical treatment and reported gradual improvement in his laboratory results and CT findings.

## 1. Introduction

Fluid collections and pseudocyst formation are the most frequent findings of pancreatitis.

Extrapancreatic fluid collections are more often detected in the lesser sac, in the anterior pararenal space, in the posterior pararenal space, in or around the left lobe of the liver, and in the spleen [1]. Extra-abdominal extension is infrequent [2] and the mediastinum is a rare site for the extension of the pancreatic secretion [1].

We report an unusual case of ascending acute exacerbation on chronic pancreatitis with mediastinal involvement detected by CT and MRI.

## 2. Case Report

A 52-year-old man came to our emergency department with a severe noncolicky pain in the right upper quadrant of the abdomen and mid-epigastrium, radiating to the back and to the retrosternum, lasting for some hours. He had also nausea and vomiting.

On clinical examination, the patient was found to be afebrile, not jaundiced, with stable vital signs.

Abdominal examination at presentation revealed a smooth abdomen, without sign of peritoneal irritation, with tenderness in the epigastrium and the right hypochondrium.

Abdominal auscultation revealed mild diminished bowel sound.

His past medical history was significant for a previous myocardial infarction. Furthermore, he had type II diabetes mellitus, combined hyperlipidemia, and gastroesophageal reflux symptoms.

His medications included acetylsalicylic acid, rosuvastatin, pantoprazole, and metformin.

There was no history of alcohol ingestion.

Laboratory tests showed an increased amylase level of 2247 mg/dL (normal, 25–125 mg/dL), an elevated lipase level of 2011 mg/dL (normal 8–78 mg/dL), an increased glycemia level of 156 mg/dL (normal 65–110 mg/dL), a higher ESR (erythrocyte sedimentation rate) of 55 mm/h (normal 1–10 mm/h), and an elevated C-reactive protein (CRP) level of 5,23 mg/dL (normal <0,50 mg/dL).

Blood count, electrolyte levels, renal function panel results, and cardiac and liver enzyme levels were in range. Admission electrocardiogram revealed normal sinus rhythm with changes suggestive of previous anterior wall myocardial infarction.

The patient was referred to radiology department for an ultrasound examination of the abdominal cavity that showed hepatic steatosis but excluded cholelithiasis, biliary sludge, and biliary duct dilatation. The pancreas was not well seen due to overlaying bowel gas.

**Figure 1:**
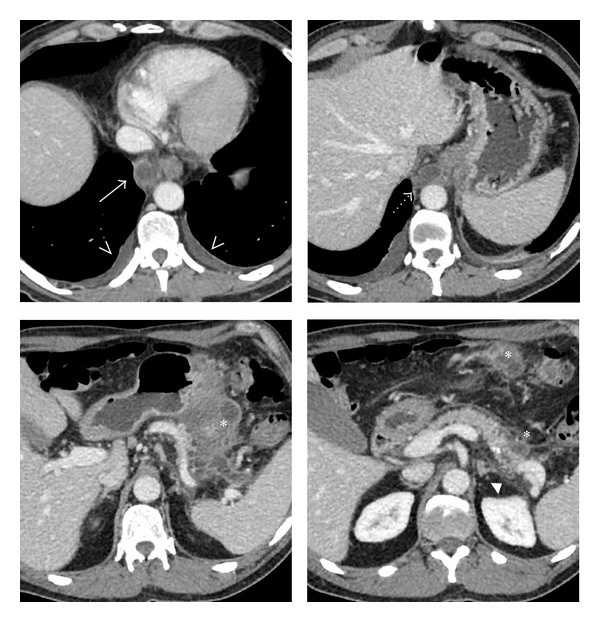
Axial images of contrast-enhanced CT show peripancreatic fluid collections and their mediastinal extension (white arrow). CT images show a cystic mass in the mediastinum, posterior to heart and right to the thoracic aorta (dotted arrow). At inferior level, fluid collections extend as a long vertical cystic lesion along the greater curvature of the stomach up to the pancreatic tail and to the greater omentum (asterisk) with increased thickening of the perirenal fascial planes (arrowhead) and stranding in the fat surrounding the fluid collections. Small amount of pleural effusion was detected in both thoracic cavities (arrowheads).

The patient had also a chest, abdominal, and pelvis CT scan that showed enlargement of the pancreatic tail with some pancreatic fluid collections (diameter about 4 cm) surrounded by a peripheral thin wall. On CT, the pancreatic fluid collections extended as a long cystic lesion along the greater curvature of the stomach up to the greater omentum (Figure 1).

CT showed the fluid collections extending also upward as a long vertical cystic lesion from the retroperitoneum to the right mediastinum, being posterior to the gastroesophageal junction, right lateral to the esophagus and thoracic aorta, and posterior to heart up to the level of left atrium (diameter about 3 cm). In this case, the close relationship between the fluid collections in the mediastinum and the esophagus suggested that the route of dissection was through the esophageal hiatus.

There were signs of peripancreatic inflammation identified as increased thickening and contrast enhancement of fascial planes (anterior renal fascia, left lateroconal fascia, and left paracolic gutter). CT revealed also changes consistent with chronic pancreatitis such as small parenchymal calcifications and mild dilatation of the pancreatic duct (3 mm) and some small lymphadenopathy and bilateral pleural effusion.

An acute exacerbation on chronic pancreatitis with extrapancreatic fluid collections spreading to the mediastinum was diagnosed. The patient was admitted to the surgical unit.

After 7 days, the patient had a contrast-enhanced MRI of the abdomen combined with MR cholangiopancreatography (MRCP) that showed normal biliary tree and excluded gallstones and/or biliary sludge.

**Figure 2:**
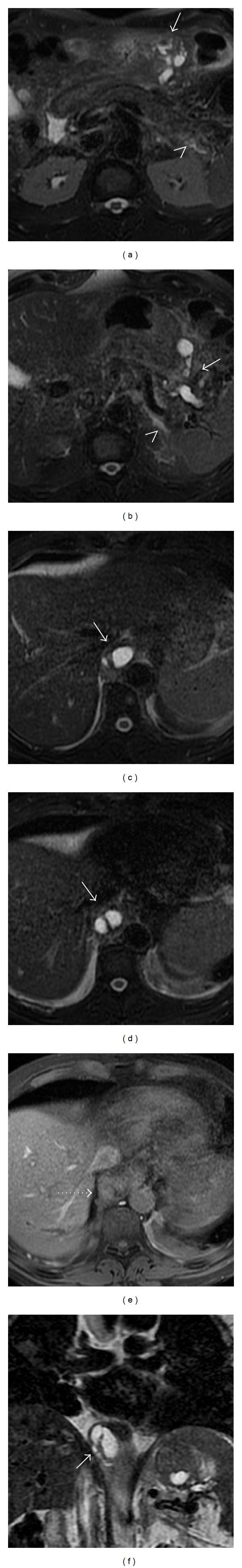
((a)–(d)) On T2-weighted fat saturated MRI, the fluid collections appear as discrete areas of homogeneous hyperintensity extending from the abdominal cavity to the mediastinum. (e) On T2-weighted fat saturated MRI, the fluid collections are slightly isointense, finding suggestive for proteinaceous or necrotic contents. (f) Coronal T2-weighted MRI image shows the fluid collections extending upward as a long vertical cystic lesion from the retroperitoneum to the mediastinum through the esophageal hiatus.

MRI showed multiple fluid collections extending from the abdominal cavity to the mediastinum that were homogeneously hyperintense on T2-weighted images and slightly isointense on T1-weighted images, with mild peripheral enhancement after administration of contrast medium (Figure 2). The isointensity of the fluid collections on T1-weighted images suggested proteinaceous or necrotic contents.

During his hospital admission, he received medical treatment, such as total parenteral nutrition, gabexate mesilate, octreotide, and ceftriaxone.

He reported gradual improvement in his symptoms and in his laboratory results.

After 40 days, patient had an amylase level of 484 mg/dL (normal, 25–125 UI/L), a lipase level of 642 mg/dL (normal 8–78 UI/L), and an ESR of 41 mm/h (normal 1–10 mm/h).

**Figure 3:**
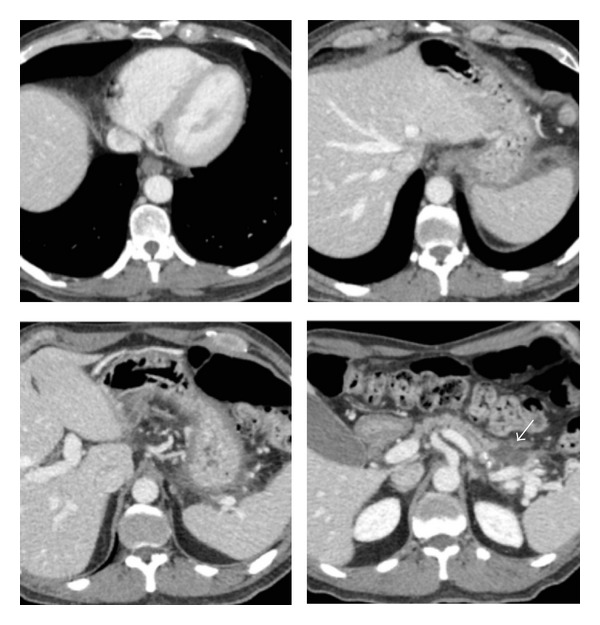
Axial images of contrast-enhanced CT obtained about five weeks after admission show a pseudocyst (3 cm) (arrow) in the pancreatic tail and the complete resolution of both mediastinal fluid collections and pleural effusion.

About five weeks after admission, follow-up chest, abdominal, and pelvis contrast-enhanced CT scan revealed the development of a fully encapsulated pseudocyst (3 cm), with a thin capsule (2 mm) and a low attenuation fluid content, in the pancreatic tail. CT scan showed also reduction of the intra-abdominal fluid collections and complete resolution of both mediastinal fluid collections and pleural effusion (Figure 3).

The patient was ultimately discharged on a low-fat diet and did have a 1-month and 3-month follow-up in the gastroenterology outpatient clinic. He is asymptomatic so far.

## 3. Discussion

We report an unusual case of an acute exacerbation on chronic pancreatitis complicated by extrapancreatic fluid collections spreading to the mediastinum detected by CT and MRI.

The fascial planes and perirenal bridging septa can be recruited to rapidly drain developing fluid collections that are formed by extravasated pancreatic secretions, debris, inflammatory exudate, fat necrosis, and hemorrhage [3].

Fluid collections spread more often along preferential drainage pathways [3], such as the lesser sac, the anterior pararenal space, the posterior pararenal space, in or around the left lobe of the liver, and in the spleen [1].

When secretions breach the thin layer of connective tissue that surrounds the pancreas, they are immediately involved with the anterior pararenal space and the lesser sac: if fluid leaks from the posterior part of the gland or from the tail, the anterior pararenal space is filled first, while an anterior perforation of the posterior layer the parietal peritoneum leads to direct extension of fluid in the lesser sac [1]. In most cases, acute extrapancreatic fluid collection distends an already existing anatomic space [1].

Thoracic involvement in pancreatic disease results from an acute disruption of the pancreatic duct on the posterior side, resulting in the leak of pancreatic secretion into the retroperitoneal space [4–8]. Once in the retroperitoneum, fluid collections track along the path of the least resistance [8]. In the majority of patients, the fluid enters the mediastinum through the aortic hiatus or the esophageal hiatus [4–9] and, thus, mediastinal fluid collections are commonly located in the posterior mediastinum [5, 6, 8].

Other less frequent sites of entry into the mediastinum are the foramen of Morgagni and the inferior vena cava hiatus, into the retrocrural space by penetrating the left diaphragm [3, 4, 6, 9]. Anterior mediastinal pseudocysts can occur from an extension through the foramen of Morgagni while middle mediastinal pseudocysts can occur through diaphragmatic erosion or an inferior vena cava hiatus [10].

Xu et al. [3] tried to identify on cadavers the possible pathways by which the peripancreatic fluid collections could spread to the mediastinum. They reported four anatomic routes: route 1 is from the peripancreatic space to the left extraperitoneal space directly and additionally into the retrocrural space via the esophageal hiatus [3]. Route 2 is from the peripancreatic space to the left extraperitoneal space via the retromesenteric plane, and further into the retrocrural space via the esophageal hiatus [3]. If the latent pathway from the peripancreatic space into the retromesenteric plane had formed, route 3 and route 4 go from the retromesenteric plane to left retrorenal plane along the left perirenal bridging septa or across the left fascial trifurcation, and they further tracked up into the retrocrural space by way of the left extraperitoneal space and esophageal hiatus [3].

In our case, the fluid collections in the mediastinum were in close relationship to the esophagus, suggesting that the route of dissection was through the esophageal hiatus.

In most of the reported cases of mediastinal extension of pancreatic fluid collections, patients had histories of previous upper abdominal trauma or surgery, alcoholism, or previous hospitalization for pancreatitis [3]. Indeed, in these cases, the more common pathways for pancreatic fluid offer greater resistance due to secondary inflammatory fibrosis from previous acute pancreatitis and, thus, the spread of fluid occurs along the path of least resistance [3]. In this case, mediastinal extension of pancreatic fluid may occur [3].

It would be interesting to speculate the cause of pancreatitis in our patient. Indeed, the patient had no history of alcohol abuse; gallstones and other abnormalities in the biliary system were excluded. Autoimmune pancreatitis was ruled out because immunoglobulin G4 fraction (IgG4) antibody was negative. Trauma was denied and other rare causes were unlikely.

We speculate that hyperlipidemia was the etiologic factor in this case, even though it has not been conclusively confirmed.

Also, our patient had no past history of pancreatitis although he had CT signs of chronic pancreatitis. On retrospective questioning and examination, the patient reported previous recurrent episodes of abdominal pain treated as gastro-oesophageal reflux. Thus, we speculate that he could have previous unrecognized episodes of pancreatitis.

Our report underlines the importance of CT and MRI findings in the diagnosis and characterization of ascending pancreatitis, thanks to evaluation of features and extension of the fluid collections provided by these two diagnostic tools.

Important in the case of ascending pancreatitis with mediastinal involvement is also the characterization of pleural effusion; indeed, pancreatic pathologies can be complicated by two types of pleural effusion [11].

The first type is associated with attacks of acute pancreatitis (in 4% of cases of mild pancreatitis and in 24% of cases of severe pancreatitis) [8, 12] and was present in our patient. This type is sympathetic in nature, usually small, generally left-sided, characterized by a normal amylase activity (below 100 U/L) and a low protein concentration (below 3 g/dL), self-limited and, thus, requiring no treatment [8, 11, 12].

The second kind of effusion is related to the presence of pancreaticopleural fistula in the course of chronic or recurrent pancreatitis, which can penetrate to the pleura, bronchi, mediastinum, or pericardium due to the gradients of pressure between the abdomen and the thorax [11]. The effusion is usually large, single-sided, and recurrent and contains a high level of amylase (usually over 1000 U/L) and proteins (over 3 g/dL) [11].

These two forms of pleural effusion should be clinically recognized, in view of their different complication rate, prognosis, and treatment [11].

A timely and accurate diagnosis of ascending pancreatitis is important due to the potentially life-threatening thoracic complications, such as mediastinal pancreatic pseudocysts, pericardial effusion, and pancreaticopleural fistulas [3, 4, 13]. Also, the extension of proteolytic enzymes through the esophageal hiatus into the posterior mediastinum, in close proximity to the heart, the esophagus, and vital vascular structures, significantly increases the potential for life-threatening complications such as penetration of the pericardium or compression of the left atrium and ventricle resulting in a right ventricular pressure gradient [14].

The treatment options of the pancreatitis are dictated by the severity of symptoms, the size of the fluid collections, the ductal anatomy, and the surgical expertise available [15].

The treatment options include medical management, drainage of fluid collection (internal and/or external), and surgery [9]. In the present case, the patient was successfully treated with conservative medical treatment. Intervention may only be indicated if infection is suspected to avoid infecting a potentially sterile collection [16].

## 4. Conclusion

Peripancreatic fluid collections are a common finding of pancreatitis and tend to extend to peripancreatic tissue, more often along preferential drainage pathways in the abdomen. In some rare cases, as the one presented in this paper, fluid collections may spread to the mediastinum leading to the formation of mediastinal collections. A prompt and accurate diagnosis of the mediastinal involvement is important due to its potentially life-threatening complications.
